# Sphingosine 1-phosphate signaling pathway in inner ear biology. New therapeutic strategies for hearing loss?

**DOI:** 10.3389/fnagi.2015.00060

**Published:** 2015-04-23

**Authors:** Ricardo Romero-Guevara, Francesca Cencetti, Chiara Donati, Paola Bruni

**Affiliations:** Department Scienze Biomediche Sperimentali e Cliniche “Mario Serio”, University of FlorenceFirenze, Italy

**Keywords:** sphingosine 1-phosphate, sensory hair-cells, inner ear neurogenesis, hearing loss, auditory neurons, neurotrophins, sphingolipids, growth factors

## Abstract

Hearing loss is one of the most prevalent conditions around the world, in particular among people over 60 years old. Thus, an increase of this affection is predicted as result of the aging process in our population. In this context, it is important to further explore the function of molecular targets involved in the biology of inner ear sensory cells to better individuate new candidates for therapeutic application. One of the main causes of deafness resides into the premature death of hair cells and auditory neurons. In this regard, neurotrophins and growth factors such as insulin like growth factor are known to be beneficial by favoring the survival of these cells. An elevated number of published data in the last 20 years have individuated sphingolipids not only as structural components of biological membranes but also as critical regulators of key biological processes, including cell survival. Ceramide, formed by catabolism of sphingomyelin (SM) and other complex sphingolipids, is a strong inducer of apoptotic pathway, whereas sphingosine 1-phosphate (S1P), generated by cleavage of ceramide to sphingosine and phosphorylation catalyzed by two distinct sphingosine kinase (SK) enzymes, stimulates cell survival. Interestingly S1P, by acting as intracellular mediator or as ligand of a family of five distinct S1P receptors (S1P_1_–S1P_5_), is a very powerful bioactive sphingolipid, capable of triggering also other diverse cellular responses such as cell migration, proliferation and differentiation, and is critically involved in the development and homeostasis of several organs and tissues. Although new interesting data have become available, the information on S1P pathway and other sphingolipids in the biology of the inner ear is limited. Nonetheless, there are several lines of evidence implicating these signaling molecules during neurogenesis in other cell populations. In this review, we discuss the role of S1P during inner ear development, also as guidance for future studies.

## Sphingosine 1-Phosphate as Sphingolipid Metabolite

Sphingolipids are a fascinating subclass of complex lipids known since a long time as key players in the correct structural organization of biological membranes. Subsequently to the understanding of their fundamental structural properties, it has been made clear that intermediates in their biosynthesis and breakdown are indeed critical signaling molecules implicated in the regulation of essential biological events. In this regard, it is presently well accepted that the vast majority of the extracellular cues involved in the regulation of cell functioning exploits at least in part sphingolipid metabolism for the accomplishment of a variety of specific biological responses.

**Figure 1:**
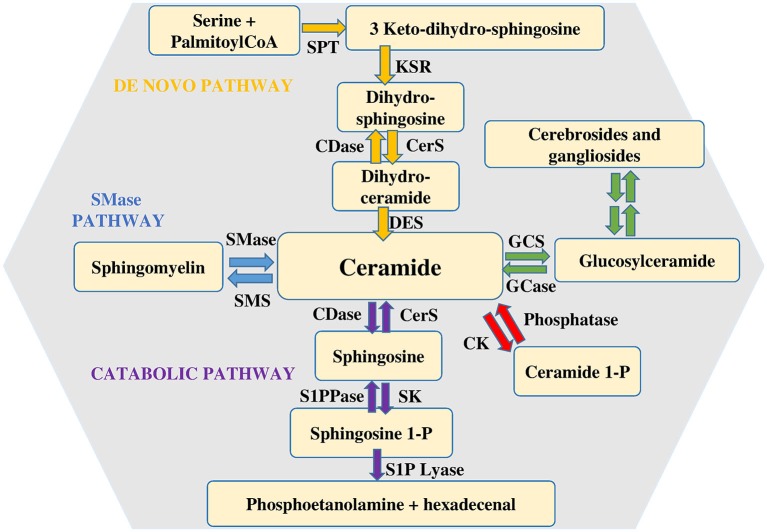
**Metabolism of sphingolipids**. Ceramide is considered the hearth of sphingolipid metabolism. It can be formed by *de novo* synthesis and then converted to other bioactive lipids. Sphingosine 1-phosphate lyase (S1P lyase) catalyzes the irreversible exit from the pathway. Abbreviations: serine palmitoyl-CoA-acyltransferase (SPT), 3-ketosphinganine reductase (KSR), (dihydro)-ceramide synthase (CerS), ceramide desaturase (DES), ceramide kinase (CK), glucosylceramide synthase (GCS), glucosyl ceramidase (GCase), ceramidase (CDase), sphingosine-1-phosphate lyase (S1P lyase), sphingosine kinase (SK), sphingosine 1-phosphate phosphatase (S1PPase), sphingomyelin (SM) synthase (SMS), sphingomyelinase (SMase).

Among the various types of sphingolipids, sphingomyelin (SM) is of paramount relevance as potential source of bioactive sphingoid molecules. Numerous reviews focus on the intricate regulation of the sphingolipid pathway (Huwiler et al., 2000; Hannun and Obeid, 2008; Figure 1). Its breakdown, catalyzed by a small family of SMases, gives rise to the formation of ceramide, an intracellular mediator *per se*, as well as a central hub for the production of other critical bioactive compounds. Ceramide is composed by a sphingoid base, named sphingosine, linked to a fatty acid via an amide bond, thus, depending on the length of the fatty acid acyl chain, various types of ceramide do exist. Another metabolic pathway that can produce ceramide is represented by its *de novo* synthesis that begins with the condensation of palmitoyl-CoA and serine catalyzed by serine palmitoyl transferase to give 3-keto-dihydrosphingosine, then reduced to dihydrosphingosine, followed by acylation reaction performed by a family of six distinct ceramide synthases, several of which are co-expressed in many different cell systems (Stiban et al., 2010; Mullen et al., 2012). The last step involves the oxidation of dihydroceramide to ceramide which is dependent on the action of a specific desaturase. Accumulation of ceramide within the cell is associated with a number of biological responses including cell growth arrest, apoptotic cell death, cell senescence, stress response making the regulation of its intracellular content critical for the fate of a given cell type (Hannun and Obeid, 2011). Once produced, ceramide can be utilized in various distinct biosynthetic pathways. The most abundant sphingolipid in plasma membrane, named SM, is generated by SM synthases in a reaction that, via transfer of phosphocholine from phosphatidylcholine onto ceramide, yields also diacylglycerol (Taniguchi and Okazaki, 2014). Alternatively, ceramide can serve as backbone in the building of glycosphingolipids, the first step being catalyzed by glucosylceramide synthase (GCS), which produces glucosylceramide, the simplest member of this family (Messner and Cabot, 2010). In turn, by addition of a galactose moiety glucosylceramide is transformed into lactosylceramide, which, by addition of one or more monosaccharides, gives raise to individual ganglioside species, recognized as vital components of membrane microdomains with a role in cell-cell recognition, adhesion, and signal transduction (D’Angelo et al., 2013). Moreover, selective phosphorylation of ceramide brought about by ceramide kinase (CK) generates ceramide 1-phosphate, a bioactive sphingolipid regarded as a powerful pro-inflammatory mediator (Gomez-Muñoz et al., 2013).

Finally, it is worth noticing that also the catabolic route by which ceramide is degraded is responsible for the production of other bioactive sphingoid compounds, among which sphingosine 1-phosphate (S1P) plays a prominent role. S1P is produced by ceramide via two specific enzymatic reactions: at first ceramidases (CDase) catalyze ceramide deacylation to sphingosine (Mao and Obeid, 2008; Ito et al., 2014), then the sphingoid base is phosphorylated to S1P by sphingosine kinase (SK). Two distinct isoforms of SK exist, designated SK1 and SK2 (Takabe et al., 2008). They are ubiquitously expressed, each contributing to intracellular S1P production. Although in some instances SK1 and SK2 can have overlapping functions, in certain cell types they have been found to differ for intracellular localization, as well as for their biological role (Maceyka et al., 2012; Neubauer and Pitson, 2013). Catabolism of S1P is also under the control of multiple enzymes. S1P lyase catalyzes the irreversible breakdown of S1P to hexadecenal and phosphoethanolamine (Kumar and Saba, 2009), while two distinct specific S1P phosphatases by removing the phosphate moiety generate sphingosine, that can be further phosphorylated to S1P or employed to feed into the so called sphingolipid salvage pathway, responsible for ceramide biosynthesis from sphingolipid breakdown (Le Stunff et al., 2002).

**Figure 2:**
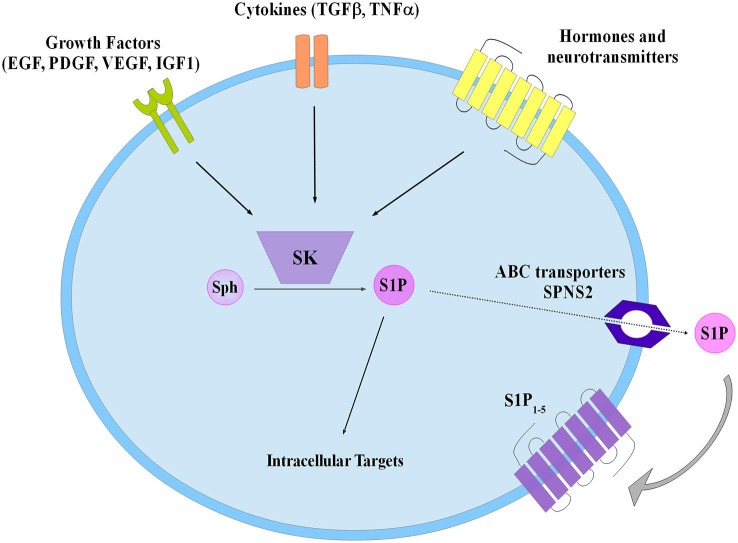
**Schematic diagram describing the inside-out signaling of S1P**. Sphingosine kinase (SK) activation by different extracellular agents leads to S1P production that both can act via intracellular targets or is extracellularly released to bind specific S1P receptors (S1P_1–5_). Transporters have been involved in S1P export such as the ATP binding cassette (ABC), and the specific spinster 2 (SPNS2).

S1P was discovered as an intracellular mediator more than 20 years ago, even though some of its intracellular targets have been identified only recently (Maceyka et al., 2012). Meanwhile, a large body of experimental evidence has been accumulated in favor of the critical role played by S1P as ligand of a family of five specific, high affinity, G protein coupled receptors named S1P_1–5_ (Ishii et al., 2004; Meyer zu Heringdorf and Jakobs, 2007). The majority of these receptors are able to activate multiple heterotrimeric G proteins, making thus possible the triggering of a wide variety of signaling pathways as well as various biological responses. Notably, S1P_1_, S1P_2_ and S1P_3_ are almost ubiquitous, while S1P_4_ and S1P_5_ expression appears to be limited to specific cell types such as those belonging to lymphoid and nervous tissue (Im et al., 2000; Kluk and Hla, 2002). Thus, taking into consideration the selective signaling downstream of individual S1P receptor subtypes, the final biological response evoked by S1P in a given cellular setting is specific, being often strictly dependent on the specific pattern of S1P receptor expressed. Exogenous S1P, mainly via ligation to one or more S1P receptors, is capable of regulating key biological processes, including cell proliferation, survival, motility as well as cell differentiation (Mendelson et al., 2014). Since ceramide and S1P are readily interconvertible lipids exerting opposite biological actions, the balance of their intracellular content is regarded as a biostat determining cell fate (Maceyka et al., 2002). Circulating plasma contains high nanomolar levels of S1P, whereas S1P availability within tissues is much lower (Schwab et al., 2005). The occurrence of such a gradient between blood and tissues is of utmost importance for the correct driving of lymphocyte and hematopoietic cell trafficking (Cyster and Schwab, 2012). Specific cell types such as erythrocytes, platelets and endothelial cells are responsible for the maintaining of the relatively high concentration of S1P in plasma (Pappu et al., 2007), although in principle every cell system can release endogenous S1P in the extracellular environment via the functioning of the widely expressed selective S1P transporter spinster-2 (SPNS2; Kawahara et al., 2009), as well as other less specific transporters such as several members of the ATP binding cassette (ABC) family (Nishi et al., 2014). Indeed, release of S1P by a given cell is regarded as a crucial step in the general mechanism by which this sphingolipid can act as paracrine or autocrine cue in the so called inside-out signaling (Figure 2). In this regard it has been clearly established that in a wide variety of cellular contexts intracellular S1P metabolism is tightly regulated by multiple extracellular agents including growth factors, hormones, cytokines and neurotransmitters and the subsequent functional interaction with S1P receptors is integral to their final biological actions (Takabe et al., 2008; Xia and Wadham, 2011; Maceyka et al., 2012). Interestingly, extensive experimental evidence has been provided for the occurrence of a complex cross-talk between S1P signaling axis and numerous different extracellular agents including: epithelial growth factor (EGF), platelet derived growth factor (PDGF), vascular endothelial growth factor (VEGF), IGF1, as well as tumor necrosis factor alpha (TNFα) and transforming growth factor beta (TGFβ), that comprises expression changes of S1P receptors and/or enzymes of S1P metabolism and/or S1P-dependent transactivation of enzyme-linked membrane receptors (Pyne et al., 2003; Takabe et al., 2008; Xia and Wadham, 2011; Maceyka et al., 2012; Donati et al., 2013a,b).

## S1P Signaling Axis in Nervous Tissue

In keeping with the essential role of S1P metabolism and signaling for correct vertebrate development, S1P biosynthesis was found to be necessary for embryonal neurogenesis. Double knockout (KO) mice for SK1 and SK2 exhibited a severely disturbed neurogenesis, including neural tube closure, defects in angiogenesis and caused embryonic lethality (Mizugishi et al., 2005). The observed neural tube defect was ascribed to the absence of S1P and the consequent increased apoptosis of neuroepithelium. The biological action of S1P was mediated at least in part by S1P_1_, given that *S1pr1* KO mice were found to display similar, even though milder, neural defects (Liu et al., 2000). A number of different studies have implicated S1P in the control of multiple biological events that occur in the various cell types present in central nervous tissue. Neural progenitor cells express S1P receptors and proliferate in response to S1P challenge (Harada et al., 2004); moreover, they are recruited by this bioactive sphingoid molecule toward a pathological area of the central nervous system (CNS) in a S1P_1_-dependent manner (Kimura et al., 2007). In this regard, S1P-mediated migration of neural progenitor cells toward an area of brain injury was found to be enhanced by antagonizing S1P_2_ (Kimura et al., 2008).

S1P is also efficacious in regulating proliferation of astrocytes (Sorensen et al., 2003; Bassi et al., 2006), specialized glial cells that by interacting with blood vessels and synapses regulate multiple aspects of brain homeostasis and functioning. Importantly, S1P was found to be secreted in response to fibroblast growth factor 2 (FGF2) identifying an autocrine/paracrine mechanism of action of this sphingolipid in the proliferation of astrocytes, with a role in the FGF2-induced cell growth signaling (Bassi et al., 2006). Since a pilot study reported that S1P induces the expression of *Fgf2* mRNA in rat astrocytes (Sato et al., 2000), it is tempting to speculate that a positive feedback loop takes place between FGF2 and S1P signaling pathway in astrocytes.

The finding that S1P_5_ is abundantly expressed in the white matter of brain (Im et al., 2001) led to the identification of this receptor subtype as predominantly present in oligodendrocytes, the myelinating cells of the CNS. In pre-oligodendrocytes S1P acting via S1P_5_ elicited process retraction, whereas it promoted survival of mature cells (Jaillard et al., 2005). However, S1P_5_ is not the unique transducer of S1P action in this cell type since S1P_1_ was reported to be up regulated by PDGF in oligodendrocyte progenitors and implicated in the growth factor-induced mitogenesis (Jung et al., 2007).

A wealth of experimental data mainly performed in hippocampal neurons support an important role of S1P receptors in the modulation of neuronal excitability as well as synapse plasticity and transmission (Kajimoto et al., 2007; Sim-Selley et al., 2009; Kanno et al., 2010; Norman et al., 2010; Kempf et al., 2014). In agreement, aberrant S1P levels and S1P receptor signaling have been reported in a range of diseases of CNS. In multiple sclerosis patients S1P concentration in cerebrospinal fluid was augmented, in accordance with the chronic inflammation status associated with this degenerative disease (Kulakowska et al., 2010). S1P concentrations were selectively decreased in the cerebrospinal fluid of adult rats in an acute and an inflammatory pain model (Coste et al., 2008). Finally, region specific S1P content decline was recently observed during the course of Alzheimer’s disease, primarily attributed to a loss of SK1 and SK2 in the hippocampus (Couttas et al., 2014).

It is important to underline that the recently developed fingolimod (FTY720) as oral therapy of multiple sclerosis directly targets S1P signaling pathway (Brinkmann et al., 2010). This compound is converted *in vivo* by SK2 into p-FTY720, that acts as high affinity agonist for all S1P receptors, except S1P_2_, and results in sequestration of lymphocytes into secondary lymphoid tissues. Besides this key mechanism of action, nonimmunological CNS mechanisms for fingolimod efficacy in multiple sclerosis therapy were identified that implicate S1P_1_ signaling in astrocytes as a key mediator, thus highlighting S1P signaling pathways within the CNS as targets for multiple sclerosis therapies (Choi et al., 2011). Intense recent studies aimed at exploring additional therapeutic applications of fingolimod, reviewed exhaustively in Brunkhorst et al. (2014), have produced very promising results that hopefully will enable the translation of some of these experimental findings into the clinics opening new avenues for the treatment of Alzheimer’s disease, cerebral malaria, neuroblastoma and neuroprotection in cranial irradiation, among others.

## An Overview of Inner Ear Morphogenesis

The mature inner ear of mammals is a remarkable structure composed by a myriad of exquisitely arranged cell types within a complex set of ducts and chambers: the vestibular system composed of the semicircular canals, the saccule and the utricle, and the auditory system formed by the cochlea, a convoluted structure where the organ of Corti (OC) resides. In this review we will emphasize the auditory system, in particular the spiral ganglion neurons (SGN), the innervation that transports the electrical stimulus from OC to the CNS. Although many questions are still open, the molecular events occurring from the initial formation of the otic placode, to the final complex tonotopic arrangement of mature SGN are starting to be understood. Several signaling pathways govern every stage in inner ear development, for example members of the FGF and WNT families are between the earliest signals triggering otic development. Later on, retinoic acid (RA) and IGF1 play an important role in the regionalization of the otic vesicle and the survival of auditory neuroblasts. Perhaps some of the most studied proteins involved in the development of SGN are neurotrophins, in particular brain-derived neurotrophic factor (BDNF) and neurotrophin 3 (NT3). The study of these neurotrophins and other signaling molecules involved in the formation and survival of SGN is also relevant from the point of view of therapeutic applications, since SGN are critical in the effectiveness of the cochlear implant.

In addition, in recent years several stem cell-based differentiation protocols have been proposed to create hair cells and sensory neurons that could be used in cell-replacement strategies (Oshima et al., 2010; Koehler and Hashino, 2014). Indeed it was recently shown that human embryonic stem cell-derived inner ear progenitors are able to restore hearing in deafened gerbils (Chen et al., 2012). It is worth mentioning that this and other protocols are developmentally-informed approaches, highlighting that a better understanding of inner ear development will be translated in more efficient differentiation protocols.

As previously mentioned, S1P signaling is functionally ubiquitous, and our group and others have shown that several cytokines, growth factors and morphogenetic cues modulate and are modulated by S1P receptors, S1P lyase and SKs.

We will briefly revisit the development of the inner and the main morphogenetic cues involved in the process and present the current knowledge on the interaction of such cues with S1P pathway in other systems. The evidence presented suggest that apart from the essential role of S1P signaling in hearing, it is worth looking at S1P pathway at earlier developmental stages.

## The Pre-Placodal Region and the Formation of the Otic Placode

Preceding the formation of the otic placode, the border region between the neural plate and the lateral ectoderm acquires the competence to respond to otic inducing signals. This region surrounding the head ectoderm called the pre-placodal domain (PPD) is established by the cooperation of bone morphogenetic protein (BMP), WNT and FGF signals emanating from the mesenchyme and the neural plate.

In *X.laevis*, the PPD marker Six1 is expressed in animal caps when the BMP inhibitors noggin and cerberus are overexpressed (Brugmann et al., 2004). In line with this, BMP blocks the expression of Six1 in its endogenous domain while noggin expands it (Ahrens and Schlosser, 2005).

In the chick Wnt8a and BMPs are expressed posteriorly and laterally to the PPD and when these pathways are blocked, the expression of the PPD markers SIX4 and EYA2 expands beyond its endogenous territory (Litsiou et al., 2005). In both works it was also demonstrated that FGF signaling was necessary to fully induce the expression of the PPD markers ectopically (Ahrens and Schlosser, 2005; Litsiou et al., 2005).

The PPD is the common ground of all cranial placodes, characterized by the expression of members of DLX and FOX families of transcription factors in several species (Quint et al., 2000; McLarren et al., 2003; Hans et al., 2004). Experiments in the chick have shown that FGF2 is able to induce the early otic marker PAX2 in explants derived from any region within the PPD but not in lateral or trunk ectoderm (Martin and Groves, 2006). Similarly in the Zebrafish *fgf8* misexpression can enlarge the size of the otic vesicle only in the regions where PPD markers have been previously expressed (Hans et al., 2004).

FGF family are also important signals that initiate inner ear development across vertebrates. In particular, the role of FGF3 during otic induction is conserved in different species. However, other FGFs such as FGF8, FGF10, FGF19 are also involved depending on the species. For example, zebrafish embryos treated with the FGF receptor inhibitor SU5402, or *fgf3/fgf8* morpholino injected do not form otic vesicles neither express the placodal marker Pax8 (Phillips et al., 2001; Maroon et al., 2002). In the chick FGF19 and FGF3 have a synergistic effect during otic placode induction (Ladher et al., 2000), although FGF2 has also been shown to induce the expression of the otic markers PAX2 and DlX3 (Martin and Groves, 2006). In mice, FGF3 together with FGF10 play a redundant role during otocyst formation, double KO animals are completely devoid of otocyst (Alvarez et al., 2003; Wright and Mansour, 2003; Zelarayan et al., 2007). In line with this, FGF receptor 2 isoform IIIb (*Fgfr2IIIb*) KO mice, the receptor isoform for FGF3 and FGF10, present gross morphological defects in the inner ear (Xu et al., 1998; Pirvola et al., 2000).

In addition to the FGF signals, WNT plays also important role during the induction and formation of the otic placode. For example, it has been reported that WNT8A induces the expression of FGF3 before the appearance of otic placode markers in the chick (Ladher et al., 2000). In murine models, conditional expression and deletion of β-catenin (CTNNB1) within the PAX2 territory increases or diminishes respectively the size of the otic domain (Ohyama et al., 2006). In agreement, overexpression of *Dkk1* gene, a WNT signaling inhibitor, impairs the development of the otic placode (Freter et al., 2008).

In summary, FGFs together with a specific level of BMP and WNT signaling inhibition first establish the PPD. Then local FGFs (e.g., FGF3 and FGF10) restrict the formation of the otic placode next to the hindbrain, which is then compartmentalized into otic and epidermis fates by the action of WNT signals.

To our knowledge, only one study has evaluated the direct involvement of S1P signaling during inner ear development (Hu et al., 2013), however there are reports indicating that S1P axis is involved in the signaling pathways that are required for the proper development of the inner ear. For example, in PC12 cells nerve growth factor (NGF) and FGF2, both important differentiation signals acting via tyrosine kinase receptors, increase the extracellular levels of S1P (Rius et al., 1997), suggesting that even different signaling molecules share in common the modulation of the S1P metabolism. Likewise, in astrocytes FGF2 can increase the activity of SK and the secretion of S1P, while S1P also induces the expression of FGF2 and phosphorylate ERK (Sato et al., 1999; Bassi et al., 2006), supporting the existence of a positive feedback regulatory loop between both signaling pathways in these cells. Analogous interaction of S1P and FGF signaling has been observed in human umbilical vein endothelial cells (HUVECs), where S1P induces the expression of VEGF, an important angiogenic factor. In these cells it was observed that the S1P-induced expression of FGF1 and its specific receptor FGFR1 preceded that of VEGF, since in cells treated with the FGFR inhibitor SU5402 or transfected with FGF1 and FGFR1 siRNA, S1P was unable to induce the VEGF expression (Chang et al., 2013). As observed in astrocytes, in HUVECs a mutual regulation between FGF and S1P signaling pathways occurs. For instance, it has been recently shown that FGF signaling is capable of regulating S1P metabolism by inducing the expression of SK1. In this system, the transcription factor KLF14, induced by FGF2, directly binds to the promoter region of SK1 gene (de Assuncao et al., 2014), establishing more clearly the mechanism of interaction between FGF and the S1P axis.

S1P plays also an important role in osteoblast differentiation. Osteoclast-conditioned medium induced the differentiation of human mesenchymal stem cells (hMSCs) towards osteoblasts. *SK1, BMP6, WNT10B* genes were highly expressed by osteoclasts and indeed when S1P signaling was blocked in hMSCs, differentiation and chemokinesis were reduced (Pederson et al., 2008). In addition, in osteoblasts it has been recently found that S1P axis increases BMP2-induced differentiation through the phosphorylation of ERK and Smad1/5/8 at a level higher than that observed following BMP2 treatment alone (Sato et al., 2012). Thus in this system S1P works as enhancer of the biological effect exerted by BMP signaling. Also in osteoblasts, S1P has been shown to interact with WNT signaling pathway by activating AKT, which inhibits GSK3β and lead to the nuclear traslocation of β-Catenin, downstream effector of WNT signaling. Indeed WNT3a-induced differentiation was impaired by the use AKT pharmacological inhibitors (Matsuzaki et al., 2013). These results and the known effects of BMP and WNT signaling in differentiation suggest that S1P could also behave as an enhancer of WNT signaling in bone formation similarly to its interaction with the BMP pathway.

## Regionalization of the Otic Vesicle and the Development of Sensory Neurons

Although morphologically uniform, the otocyst is compartmentalized by the expression of otic markers allocated at different regions within the otocyst. Members of Notch, FGF, RA and SHH signaling pathways are involved in this process.

Although *Fgf3/Fgf10* double KO mice lack completely otic vesicle, the effect of single mutation affects only the expression patter of individual markers, such as DLX5 and PAX2, within the vesicle (Wright and Mansour, 2003). Indeed FGF3 and FGF10 are also expressed at different territories in the otocyst (Pirvola et al., 2000), suggesting that FGFs are important in otocyst regionalization.

RA is a morphogen with a known role in otic vesicle regionalization. In chick and mouse it was found that RA synthesis and degradation are allocated to the posterior and anterior ectoderm respectively, creating in this manner a RA gradient in the otic vesicle. Indeed exposure to RA posteriorise the entire otocyst, reducing the size of the prosensory domain, the area where sensory cells arise (Blentic et al., 2003; Bok et al., 2011). In the zebrafish RA expands anteriorly the expression of the transcription factor Tbx1 normally restricted to the posterior otic vesicle. This leads to increased Notch activation and reduction of the sensory domain (Radosevic et al., 2011).

RA also controls the expression pattern of FGF3 and FGF10, which may underlie the development of the sensory domain (Cadot et al., 2012; Economou et al., 2013). In this regard, it has been shown that FGF10 expression precedes the proneural genes *Neurog1* and *Neurod1* in the otocyst, and FGF10 overexpression increases the number of neuroblasts that delaminate (Alsina et al., 2004).

Sonic hedgehog (SHH) is another signaling molecule that participates in the formation of the otocyst ventromedial domain. *Shh* KO mice fail to maintain the expression of PAX2, OTX2, in the otocyst neurogenic region. As a consequence, ventral structures such as the cochlea and the cochlear ganglion never form (Riccomagno et al., 2002; Bok et al., 2007).

As early as E9 in the mouse, sensory neuroblast start to delaminate from the ventral neurogenic domain of the otocyst, the process continues until E14. Notch, neurotrophins and IGF1 are important in the differentiation and survival of neuroblast. Notch pathway has several functions, it restricts the non-neuronal domain of the otic vesicle posteriorly, and in the other hand, by mechanism of lateral inhibition limits the number of otic neuroblast that delaminate (NEUROD1+ cells) (Haddon et al., 1998; Abelló et al., 2007). In general, it has been observed that Notch signaling activation is necessary to the formation of sensory cells (Kiernan et al., 2006; Daudet et al., 2007). Indeed, the cell decision between neurogenic vs. sensory is regulated by Notch as NEUROG1+ cells restrict neighbor cells of adopting a neural fate but maintains them as sensory precursors (Raft et al., 2007). As neuroblasts (NEUROG1+) continue their way out of the otocyst, another transcription factor NEUROD1 is switched on. These genes are considered to be essential in the formation of the spiral ganglion. *Neurog1* and *Neurod1* KO mice lack completely innervation to the cochlea which appears morphologically normal (Ma et al., 2000; Kim et al., 2001). However, *Neurod1* mutants form sensory neurons but these do not mature and undergo apoptosis. The expression of the neurotrophin receptors TRKB and TRKC failed in these animals, explaining the increased cell death.

Neurotrophins are important survival factors for many neuronal populations. The inner ear expresses two of them, BDNF and NT3 which are essential for the development of the SGNs. Double KO mice for either *Bdnf/Nt3* or their receptors *TrckB/TrckC* are devoid of cochlear innervation (Ernfors et al., 1995; Silos-Santiago et al., 1997). Their expression pattern forms a gradient for the topographic organization of the sensory neurons (Pirvola et al., 1992; Fariñas et al., 2001). For example BDNF expression starts at the apex of the cochlea at E12.5 and progresses towards the base as the hair cells mature while NT3 has an inverse base-to-apex expression pattern (Schimmang et al., 2003; Sugawara et al., 2007). In fact, exposure to one or the other changes the behavior of SGN towards apical or basal neurons regardless of their position (Adamson et al., 2002). It has been observed that BDNF and NT3 are able to differentially regulate important electrophysiological proteins such as AMPA receptors and synaptophisin (Flores-Otero et al., 2007).

There are other cues involved in the formation of the spiral ganglion. FGFs have been shown to influence the migration and neurite outgrowth of inner ear neuroblasts. In chick otocyst explants, the expression of the BDNF receptor, TRKB is induced by the treatment with FGF2, together with this change, neural progenitor migration and the axon outgrowth accelerates (Brumwell et al., 2000).

IGF1 also plays a role in the formation of mature spiral ganglion cells. *Igf1* KO mice are profoundly deaf by the third postnatal week. Although the cochlea seems normal at birth in these mice, the maturation of several structures of the inner ear such as the tectorial membrane and the spiral ganglion is impaired during the second and third postnatal week (Camarero et al., 2001). A marked apoptosis of SGNs, decreased soma size and an immature synapsis with the OC were observed in *Igf1* KO mouse. Thus IGF1 is prescindible for normal development but it is later required for the maturation and survival of spiral ganglion cells at postnatal stages. Nonetheless examination of the inner ear of *Igf1* KO at earlier developmental stages is still missing. In chick, IGF1 is necessary for the proliferation and further differentiation of auditory neuroblasts (Camarero et al., 2003). The biological effect of IGF1 in this model has been shown to be mediated by the activation of PI3K-AKT signaling pathway (Aburto et al., 2012).

Compelling evidence in several cellular settings models highlights the involvement of S1P axis in the signaling pathways discussed before. In human breast cancer cell lines for example, RA has shown an anti-proliferative effect mediated upon binding to the RA receptor RARα, leading to overproduction of ceramide and downregualtion of SK1. On the contrary, in cells transfected with a dominant-negative form of RARα, RA-induced growth inhibition is hampered, being accompanied by the increased expression and activity of SK1 (Somenzi et al., 2007). On the other hand S1P metabolism affects RA signaling: for example in colon cancer cells, S1P down-regulates the receptor RARβ, thus making cells refractory to RA-induced growth inhibition (Sun et al., 2012).

The cross-talk between neurotrophins and S1P metabolism is also well established. In PC12 and dorsal root ganglion cells, neuritogenesis induced by NGF depends in the membrane translocation of SK1 and the activation of S1P_1_, while S1P_2_ and S1P_5_ have an inhibitory role in neurite formation (Toman et al., 2004). This underlines the importance of understanding the function of this pathway in a particular cell type, since different S1P receptors can trigger disparate biological functions.

In a mouse model of Rett syndrome, *Mecp2* KO, the S1P agonist p-FTY720 showed effectiveness in improving the symptoms of the disease, correlated with increased BDNF levels in several neuronal populations (Deogracias et al., 2012). In another study using cortical neurons p-FTY720 also induced the expression BDNF which protected cells against oligomeric amyloid-β toxicity in an *in vitro* model of Alzheimer disease (Doi et al., 2013). In oligodendrocytes, NT3-induced survival was found to be dependent upon SK expression and translocation (Saini et al., 2005).

Apart from differentiation and survival, it was demonstrated that NGF increased the number action potentials in sensory neurons through a SK1-dependent mechanism (Zhang et al., 2006), even S1P treatment alone was enough augment the number of action potentials in these cells. Thus, it seems that the modulation of S1P metabolism is common molecular mechanism to transmit several of the biological effects elicited by neurotrophins in diverse neuronal populations.

As regards the interaction of S1P with IGF1 signaling, it is important to recall that our laboratory recently found an involvement of S1P axis in IGF1 signaling. We proved that the myogenic effect of IGF1 in skeletal muscle cells is dependent on the activity of SK1 and SK2; if these kinases are blocked by pharmacological inhibitors or siRNA down-regulation, the expression of the myogenic markers myogenin and caveolin-3 is reduced (Bernacchioni et al., 2012). In the ear, IGF1 is currently being studied as a potential polypeptide to be used in the treatment of sensorineural hearing loss and the first results of a clinical trial involving 25 patients with sudden sensorineural hearing loss are very promising (Nakagawa et al., 2014; Yamamoto et al., 2014). Thus, from the point of view of medical application, it would be very interesting to investigate the potential involvement of S1P axis in IGF1 signaling in the context of the inner ear, and explore the possibility of using S1P or S1P receptor agonists/antagonists to prevent hearing loss.

In summary, the formation of the inner ear is a complex and dynamic process that involves the integration of signals such as WNTs, FGFs, RA, SHH and Notch ligands. The recognized interaction between these extracellular cues and S1P in other cell systems should prompt to study its possible involvement in the inner ear. This would shed light not only in the understanding of basic development of the inner ear, but also in the translation of more robust differentiation protocols to generate sensory cells *in vitro* from stem/progenitor cells.

## The Role of Sphingolipid Signaling in the Inner Ear

It is still not clear the precise role of different sphingolipids in the biology of the inner ear, but there are several reports that highlight their importance and make them attractive targets that deserve further investigation.

For example it was recently found that the simplest ganglioside named GM3, generated by addition of a sialic acid to lactosylceramide in a reaction catalyzed by GM3 synthase, plays an important role in inner ear physiology. GM3 synthase (*St3gal5*) KO mice have been shown to be profoundly deaf due to specific deterioration of the OC (Yoshikawa et al., 2009). The hearing loss started as early as P14, progressing rapidly and by P17 KO mice were profoundly deaf with a substantial loss of hair cells. The molecular mechanism responsible for hearing loss was not determined. Although gangliosides GM3 and GT1b were expressed in the OC, SGN and stria vascularis (SV), no changes in K^+^ and endocochlear potential (EP) were observed in the KO mice (Yoshikawa et al., 2009). Noteworthy, in rat organotipic cultures gangliosides GM1 and GM3 were shown to diminish gentamicin-induced hair cell death (Nishimura et al., 2010). In addition, in the *St3gal5* mutants there was an accumulation of other ganglioside species, whether these can induce apoptosis is unknown. In this regard, GM3 synthase deficient human fibroblasts showed increased apoptosis and accumulation of these ganglioside species (Liu et al., 2008). An alternative explanation could be that GM3-derived gangliosides could modulate growth factor response necessary for the maturation and survival. Indeed, in the work of Liu et al. fibroblasts of such patients have reduced EGF-induced proliferation and migration (Liu et al., 2008). It is important to mention that one of these patients was deaf (Fragaki et al., 2013).

Sphingomyelin, the most abundant sphingolipid in biological membranes, has also been demonstrated to be involved in hearing and maintenance of inner ear structures such as SV and hair cells and the EP. The SM synthase-1 (*Sms-1*) KO mouse presents hearing loss although this is milder than the one observed in *St3gal5* KO mouse and other mutant mouse strains discussed later, *Spns2* and *Sp1r2* KOs, and affects mainly the low and middle frequency hearing (Lu et al., 2012). These mice have a decreased EP compared with wild type (WT) of the same age, but the drop was not as robust as the ones observed in the *St3gal5*KO for example, perhaps accounting for the milder phenotype. Together with the SV defects and the decreased EP, there was an aberrant expression of the K^+^ channel KCNQ1 in KO mice. However this was probably a secondary consequence, as its expression in the SV was only assessed at 3 months of age and the K^+^ concentration was found to be equal at 1 month between KO and WT littermates.

Finally, only recently S1P signaling emerged as an important pathway in the development of the inner ear and there are only few reports describing its role. For instance, explants cultures of rat cochlea have shown that gentamicin-induced hair cell death can be reduced upon the addition of S1P or increased by the presence of ceramide in the culture media, suggesting that hair cells could respond to these sphingolipids (Nishimura et al., 2010). Moreover, a recent work provides some details on the molecular mechanism that is responsible for the protective effect of S1P on hair cells. Indeed, S1P_1–3_ were found to be expressed in rat cochlea, both in spiral ganglion and OC, by non quantitative RT-PCR analysis and more interestingly an S1P_2_ antagonist was capable to augment gentamicin-induced hair cell loss, pointing at a role of this receptor in inner ear cell survival (Nakayama et al., 2014). Therefore it is not clear if the effect of ceramide and S1P may also occur indirectly by a secreted signal released from supporting cells, where for example S1P_2_ has been observed (Herr et al., 2007). Nonetheless, the insights could have an important implication for the use of these metabolites as otoprotective agents. In addition, it remains to be addressed if S1P and ceramide content change after ototoxic damage *in vivo* in the cochlea, this could shed new light into the role of these sphingolipids during hair cell degeneration.

Single *S1pr2* and double *S1pr2/S1pr3* KO have been shown to be profoundly deaf by the third postnatal week (MacLennan et al., 2006; Herr et al., 2007; Kono et al., 2007). Histological examination of the mutants at 6 weeks after birth shows a normal cochlear structure devoid of hair cells and auditory neurons. Kono et al. suggested that the loss of auditory function was caused by defects in the SV, a highly vascularized structure that maintains the EP. Indeed, well before the loss of hair cell and neurons, at postnatal week 2, a thickened SV with aberrant capillaries morphology and defects in the marginal cell boundaries could be observed (Kono et al., 2007). However, K^+^ concentration and the EP were not measured in these mutant mice. Thus, the loss of hair cells could not be completely attributed to an altered EP. Recently, the S1P specific transporter SPNS2 was found to be involved in audition, as mutant mice showed a gradual and profound hearing loss starting from the second postnatal week (Chen et al., 2014). Similarly to the *S1pr2* KO mice, *Spns2* mutants showed defects in the SV characterized by thicker capillaries with increased branching, as well as abnormal marginal cell borders. These defects and a decrease in the EP were the earliest abnormalities observed in the *Spns2* KO (P14). Thus, it was concluded that SPNS2 is necessary to maintain the function of the SV and the EP, while the loss of hair cells and the downregulation of KCNQ1, KCNJ10, GJB2 and GJB6, known to maintain the EP, was secondary. This work is in agreement with the results obtained in the *S1pr2* KO mice (Kono et al., 2007), and suggests an involvement of the S1P-SPNS2-S1P_2_ axis in the maintenance of the structure of the SV. However, how the S1P signaling maintains the EP is not clear, indeed, in the *Spns2* KO mice no permeability between perylimph and endolymph compartments, neither leakage from the capillaries of the SV was observed in the mutants (Chen et al., 2014). In this regard, S1P has shown to increase the excitability of cardiac fibroblast by activating the K^+^ channel KIR6.1 in a S1P_3_-dependent manner (Benamer et al., 2011) or increase the firing rate of rat hippocampal neurons (Norman et al., 2010), suggesting that this pathway can regulate electrophysiological properties in diverse cell types. Another open question that deserves further investigation is the possible involvement of other S1P transporters in the inner ear. For instance in the *S1pr2* KO mice, a vestibular phenotype was observed while in the *Spns2* mutant only hearing function was disrupted. In this regard, it is unknown if in the vestibular system other S1P transporters may substitute the function of SPNS2. Only the MRP1 transporter has been found in the auditory and vestibular system and although it is known to transport S1P in other systems (Mitra et al., 2006), no vestibular phenotypes has been reported in the KO mice (Zhang et al., 2000). Other non-specific S1P transporters have been found in the mice sensory epithelia such as ABCG2 (Savary et al., 2007), but it is not known if it has role in regulating the extracellular levels of S1P. In fact, it is also unknown if the local release of S1P is necessary to maintain the EP as S1P has not been measured in the cochlea of WT or in *Spns2* KO mice.

In addition, a direct involvement of the S1P signaling in the maintenance of hair cells separate from the EP cannot be ruled out until cell type specific mutants become available. In this regard there are some indications that S1P signaling may have a direct role in sensory cells, evidenced for example by the localized expression of SPNS2 in outer hair cells (Chen et al., 2014), S1P_2_ expression in supporting cells and S1P_3_ in that area of SGN (Herr et al., 2007). Even more, the zebrafish ortholog of mammal *S1pr2*, miles-apart (*mil*) has been expressed during developmental stages at positions where the neuromast develop. In addition *mil* morphants have a reduced number of neuromasts in the lateral line while the mRNA transfected embryos have an excess of neuromasts. Equally, morphants and mRNA transfected animals presented patterning defects in the otic vesicles (Hu et al., 2013). In summary, at least in the zebrafish S1P_2_ is involved in the formation of the otic vesicle and hair cells. If it has a similar role in the mouse has not been studied yet although the gross morphology of the cochlea and the hair cells is normal at birth in the* S1pr2* KO mice.

Altogether these works indicate that sphingolipids are essential for normal hearing function, but their molecular targets are still to be discovered either in the sensory epithelium itself, the spiral ganglion and importantly, in the SV, that shows some of the earliest defects in the KO models. The results described before are summarized and presented in Table 1. Another important aspect to be explored in the future will be the relationship between different categories of sphingolipids, using cell lines derived from these animal models. Those studies will help us to pinpoint if there is a common mechanism involving hearing loss in the models described before. Such a task will be accomplished further with the help of cell lines with selective deletion of enzymes and receptors involved in S1P signaling axis, and possibly by using iPS-derived cells to uncover the role of S1P signaling during the differentiation of inner ear sensory cells.

**Table 1:** **Knockout models for genes involved in sphingolipid metabolism and signaling have been found to present inner ear defects**.

Gene	Model	Phenotype and function	References

Sphingosine 1-phosphate receptor 2 *(S1pr2)* Alternative names: *Edg5, H218, LPb2, S1P2, Gpcr13*	Mouse	At 1 month of age there was a profound hearing loss together with a decreased number of hair cells, at 4 months spiral ganglion neurons were completely absent as well. At P14 a thickened stria vascularis with disorganized marginal and basal cells was observed as well as thick vessels with excessive branches.	Kono et al. (2007)
		Approximately 40% of 2 months old KO mice displayed a vestibular phenotype, while invariably all mice were deaf from P22, the onset of degeneration of the Organ of Corti, characterized by an abnormally thin stria vascularis, loss of hair cells and a striated tectorial membrane.	MacLennan et al. (2006)
Sphingosine 1-phosphate receptor 3 *(S1pr3)* Alternative names: *Edg3, Lpb3, S1p3,* *AI132464*	Mouse	In combination with *S1pr2* knockout (KO), there was a progressive loss of hair cells and spiral ganglion neurons starting at 1 month of age.	Herr et al. (2007)
		The double KO *S1pr2* and *S1pr3* showed in addition to hearing loss, a vestibular phenotype not present in the single KO *S1pr2*.	Kono et al. (2007)
Sphingosine 1-phosphate receptor 2 *(s1pr2)* Alternative names: *mil, edg5, s1p2*	Zebrafish	Downregulation or overexpression of *s1pr2* gene reduced the size of the posterior otolith, altered its morphology at 48 h postfertilization and later, at 72 h disturbed the formation of the semi-circular canals. In addition, there was a decrease in the number of lateral line neuromast or these were shrunken with less hair cells and a higher proportion of apoptotic cells at 6 days postfertilization. Conversely *s1pr2* mRNA injection increased the number of posterior neuromast and the number of hair cell within the neuromast.	Hu et al. (2013)
Spinster homolog 2 *(Spns2)*	Mouse	Progressive hearing loss started at P14 and was almost complete by the third postnatal week, robust drop in the endocochlear potential was observed during this window followed by hair cell loss at 1 month of age. Other defects included an excessive branching of capillary net and reduced number of marginal cells in the stria vascularis, no vestibular problems were observed.	Chen et al. (2014)
Sphingomyelin synthase 1 *(Sgms1)* Alternative names: *Mob, Sms1, Sor1, C80702,* *Tmem23, AI841905,* *9530058O11Rik*	Mouse	Auditory brain response analysis from 1 month of age onwards showed that the low and middle frequency hearing regions were the most affected. In line with the phenotype, there was a drop of approximately 20 mV in the endocochlear potential together with a thin and shortened stria vascularis in the mutants. Within the stria vascularis, the marginal cells look disorganized.	Lu et al. (2012)
ST3 beta-galactoside alpha-2, 3-sialyltransferase 5 *(St3gal5)* Alternative names: *3S-T, Siat9, GM3 synthase*	Mouse	Hearing loss evident at P14, selective degeneration of the organ of Corti while the rest of the cochlear structures were morphologically normal as well as the endocochlear potential.	Yoshikawa et al. (2009)

## Conclusions and Remaining Questions

Overall, the here reported experimental findings highlight the crucial role of S1P signaling axis in inner ear biology. However there are several remaining questions that deserve further investigation. The different animal models discussed here support that S1P signaling is necessary during inner ear maturation and hearing at postnatal age in mice. Nonetheless, to our knowledge only one study in the zebrafish has assessed the involvement of this pathway during development (Hu et al., 2013). Thus, it remains to be seen if deletion of S1P receptors or S1P-metabolizing enzymes in mice affect gene expression in the inner ear at early developmental stages.

Importantly, it is still necessary to investigate in more detail the molecular mechanism by which S1P axis is regulating inner ear function in postnatal age, such as the modulation of growth factor receptors, the expression of K^+^ channels, critical for the EP, as well as the activation of prosurvival pathway in hair cells and sensory neurons. This is important and consistent with the variety of biological effects elicited by S1P in diverse cell types.

Future studies will confidently clarify the molecular mechanisms that regulate S1P metabolism and S1P receptor expression in the various aspects of inner ear sensory cell biology and hopefully they will disclose the therapeutic potential of S1P signaling pathway in hearing loss.

## Conflict of Interest Statement

The authors declare that the research was conducted in the absence of any commercial or financial relationships that could be construed as a potential conflict of interest.
